# Effect of Microparticle Crystallinity and Food Matrix on the Release Profile and Antioxidant Activity of Encapsulated Gallic and Ellagic Acids During Simulated In Vitro Intestinal Digestion

**DOI:** 10.3390/antiox14101211

**Published:** 2025-10-07

**Authors:** Yesica Vilcanqui, Alejandra Quintriqueo-Cid, Patricio Romero-Hasler, Begoña Giménez, Eduardo Soto-Bustamante, Paz Robert

**Affiliations:** 1 Departamento de Ciencia de los Alimentos y Tecnología Química, Facultad de Ciencias Químicas y Farmacéuticas, Universidad de Chile, Dr. Carlos Lorca Tobar 964, Independencia, Santiago 81380494, Chile; yvilcanquic@unam.edu.pe (Y.V.); aquintriqueo@ug.uchile.cl (A.Q.-C.); patricio.romero@ciq.uchile.cl (P.R.-H.); 2 Departamento de Ciencia y Tecnología de los Alimentos, Facultad Tecnológica, Universidad de Santiago de Chile, Av. Víctor Jara 3769, Estación Central, Santiago 9170124, Chile; bego.gimenez@usach.cl; 3 Departamento de Química Orgánica y Fisicoquímica, Facultad de Ciencias Químicas y Farmacéuticas, Universidad de Chile, Dr. Carlos Lorca Tobar 964, Independencia, Santiago 81380494, Chile; esoto@ciq.uchile.cl

**Keywords:** microencapsulation, gallic acid, ellagic acid, spray-drying, digestion, antioxidant activity

## Abstract

The development of phenolic-based functional food ingredients is of growing interest due to their beneficial effects on human health. This study investigated the combined influence of microparticle physical state, phenolic compound type (gallic acid, GA; and ellagic acid, EA), and model food matrix on the release profile, bioaccessibility, and antioxidant activity of GA and EA during in vitro gastrointestinal digestion. GA and EA were encapsulated with inulin (In) by spray-drying. By varying formulation and operational conditions, both semicrystalline (GA-InSc, EA-InSc) and amorphous (GA-InA, EA-InA) microparticles were obtained. Microparticles were characterized for crystallinity, encapsulation efficiency, particle size, morphology, and release profile during in vitro simulated gastrointestinal digestion following the INFOGEST method. The physical state of microparticles and type of phenolic compound critically influenced release profile, bioaccessibility, and antioxidant activity during digestion. GA, being more water-soluble, was rapidly released, reaching nearly 100% in the gastric phase, whereas EA exhibited limited gastric release and higher intestinal release, particularly in EA-InSc. Incorporation into different food matrices further modulated these effects; carbohydrate- and blend-based matrices improved phenolic release and antioxidant activity for both compounds. These findings highlight the importance of microparticle formulation, phenolic characteristics, and matrix interactions in designing functional food ingredients with optimized health benefits.

## 1. Introduction

Phenolic compounds have attracted considerable attention for their potential as functional ingredients in the food, nutraceutical, and pharmaceutical industries. However, despite their well-recognized health benefits, their practical application in the food sector is still restricted by issues related to stability, aqueous solubility, and bioavailability [1].

Ellagic acid (EA) and gallic acid (GA) are phenolic compounds that have demonstrated multiple biological activities, including antioxidant, anti-inflammatory, and anticancer effects [2,3]. EA is present in fruits such as pomegranates and strawberries, as well as in seeds and nuts, and occurs in free form, as glycosides, or as ellagitannins [3]. EA exhibits poor water solubility (10 µg/mL) and limited bioavailability; it is only partially absorbed in the small intestine and is further metabolized by the gut microbiota in the colon to produce urolithins [3]. In contrast, GA is widely distributed in tea, grapes, berries, and fruits. Unlike EA, GA exhibits much higher water solubility (10,000 µg/mL) and is more efficiently absorbed in the small intestine [4].

However, phenolic compounds such as EA and GA are highly susceptible to degradation under environmental factors (temperature, oxygen, light, humidity, and pH), food processing conditions, and gastrointestinal factors (pH, salts, enzymes), leading to the reduction or loss of their functional properties [5]. To overcome these drawbacks, microencapsulation has been recognized as an effective approach, as it enables the confinement of phenolic compounds within a protective matrix, thereby enhancing their stability during processing, storage, and digestion [5,6]. Several encapsulation methods have been reported for EA and GA, including spray drying [6,7,8], freeze drying [7], electrospinning [9], ionic gelation [10], ionotropic gelation [11,12], emulsion-based methods [13], liposomes [14], and molecular inclusion [15]. The selection of an encapsulation technique is influenced by multiple factors, including cost, equipment availability, process complexity, and desired particle size. Among these, spray-drying is one of the most widely used methods for microencapsulation due to its low cost, scalability, and ability to protect heat-sensitive compounds such as EA and GA [6]. Nevertheless, encapsulation efficiency strongly depends on the type of biopolymer employed as the encapsulating agent.

Inulin is an attractive biopolymer for encapsulating phenolic compounds due to its favorable characteristics: it is moderately water-soluble, exhibits low viscosity, and is colorless, which makes it particularly suitable for spray-drying applications [16]. In addition, inulin exerts prebiotic effects that promote the growth of beneficial gut bacteria [17] and thereby contribute to gut health. Furthermore, inulin can generate microparticles in either amorphous or semicrystalline states [18]. Several factors have been reported to influence inulin crystallinity, including spray-drying conditions (inlet air temperature, infeed temperature) [18], addition of antisolvents (ethanol, methanol, acetone, and n-propanol) [19], and incorporation of crystalline seeds into the infeed solution [20]. The physical state of inulin (amorphous or semicrystalline) plays a decisive role in determining the physicochemical characteristics of the microparticles, their stability during storage, and the release behavior of the encapsulated phenolic compounds throughout digestion [21].

To reproduce human physiological conditions, in vitro gastrointestinal digestion models are frequently used. Among them, the INFOGEST protocol is a static method that sequentially simulates the oral, gastric, and intestinal phases [22]. This protocol has been validated for its reproducibility and applicability in studies with food matrices [1]. A previous work has applied in vitro digestion to assess the bioaccessibility of phenolic compounds and in microparticles loaded with phenolics from *Bougainvillea glabra* bract extracts using semicrystalline inulin [23]. However, to the best of our knowledge, no studies have evaluated the effect of microparticle physical state (amorphous vs. semicrystalline) when incorporated into a food matrix and subjected to simulated digestion. Therefore, the present study aims to investigate the combined influence of microparticle physical state, phenolic compound type (EA vs. GA, differing in solubility), and model food matrix on the release profile, bioaccessibility, and antioxidant activity of EA and GA during in vitro simulated gastrointestinal digestion.

## 2. Materials and Methods

Ellagic acid (EA, ≥98% purity) was obtained from AK Scientific (California, CA, USA), and gallic acid (GA, ≥98% purity) was purchased from Sigma-Aldrich (Santiago, Chile). Inulin Orafti^®^ HP (DP ≥ 23) was purchased from Blumos S.A. (Santiago, Chile). Solvents and reagents, analytical-grade and HPLC-grade, were acquired from Merck (Santiago, Chile). The food matrices were sucrose, pectin (Quimatic, Santiago, Chile), maltodextrin (Prinal, Santiago, Chile), concentrated whey protein (Prinal, Santiago, Chile), and sunflower oil (Natura, Aceitera General Deheza S.A., Buenos Aires, Argentina). The digestive enzymes pepsin from porcine gastric mucosa (P6887, 3200 AU/mg), pancreatin from porcine pancreas (P7545, 8 × USP specifications), lipase from porcine pancreas (L3126), and porcine bile extract (B8631) were obtained from Sigma-Aldrich (Santiago, Chile).

### 2.1. Central Composite Design for the Preparation of Ellagic Acid-Inulin Microparticles Using Ethanol as Crystallinity Inducer

Experimental design: the encapsulation of EA with inulin, using ethanol as a crystallinity inducer, was performed according to a central composite design with axial points (12 runs: 4 experimental, 4 axial, and 4 central points). The independent variables were the inlet air temperature during spray drying (T_e_: 120–180 °C) and the ethanol percentage in the infeed dispersion (EtOH, 10–40%). The dependent variables were the crystallinity index (CI) and the encapsulation efficiency (EE) of the EA. Response surface methodology was applied to evaluate the effects of ethanol concentration and T_e_ on CI and EE of EA. Analysis of variance (ANOVA), coefficient of determination, and lack of fit tests were carried out using Statgraphics software (Program 7.0, Manugistics Inc., Rockville, MA, USA).

The experimental data were fitted to a second-order regression model represented by Equation (1).

(1) $$Y=\beta_{0}+\sum_{i=1}^{2}\beta_{i}X_{i}+\sum_{i=1}^{2}\beta_{ii}X_{i}^{2}+\sum_{i=1}^{1}\sum_{j=i+1}^{2}\beta_{ij}X_{i}X_{j}+\varepsilon$$

where *Y* was the response (EE or CI); *β*_0_ was the intercept term; subscripts i and j ranged from 1 to 2 (number of variables); *β_i_* values were the linear coefficients; *β_ii_* values were the quadratic coefficients; *β_ij_* values were the cross-product coefficients; *X_i_* and *X_j_* were the levels of the independent variables; and *ε* was the error term.

A multiple optimization analysis based on the desirability function was subsequently performed. CI and EE were maximized to obtain semicrystalline EA-inulin microparticles (EA-InSc), whereas CI was minimized and EE was maximized to obtain amorphous EA-inulin microparticles (EA-InA). Finally, the optimal conditions determined for EA-InSc and EA-InA were then applied to prepare GA-inulin microparticles in both semicrystalline (GA-InSc) and amorphous (GA-InA) forms.

Infeed dispersion preparation: Inulin (15% *w*/*w*) was dispersed in distilled water under agitation and heated to 90 °C with continuous stirring (500 rpm) until completely dissolved. The solution was subsequently cooled to 20 °C at a controlled rate of 1.4 °C/min using a jacketed beaker connected to a recirculating bath (JSRC-13C, JS Research Inc., Gongju, Republic of Korea). EA (0.150 g), previously dispersed in ethanol (ranging from 10% to 40%), was added to the inulin dispersion and stirred for 30 min at 20 °C. The resulting dispersion was fed into a mini spray dryer (B–290, Büchi, Flawil, Switzerland) under the following drying conditions: T_e_ ranging from 120 °C to 180 °C, infeed temperature of 20 °C, feed rate of 1 mL/min, airflow of 600 L/h, and atomization pressure of 20 psi. The microparticles obtained were stored in amber containers protected from light at −20 °C until further analysis.

According to the multiple optimization analysis (desirability function), the optimal conditions for producing semicrystalline EA-inulin microparticles (EA-InSc) were an ethanol concentration of 36.5% and a T_e_ of 114 °C. For amorphous EA-inulin microparticles (EA-InA), the optimal conditions were an ethanol concentration of 6.8% and a T_e_ of 148 °C. GA-inulin microparticles in amorphous (GA-InA) and semicrystalline (GA-InSc) forms were prepared under the same conditions established for EA.

### 2.2. Characterization of Ellagic Acid- and Gallic Acid-Inulin Microparticles

The four microparticle systems (EA-InSc, EA-InA, GA-InSc, and GA-InA) were characterized.

#### 2.2.1. Crystallinity Index (CI)

X-ray diffraction patterns were obtained using a SAXSPoint 2.0 WAXS/SAXS system (Anton Paar, Graz, Austria) equipped with a Primus 100 microfocus X-ray source with Cu Kα = 1.54178 Å), operating at 50 kV and 100 µA. The beam was collimated using an ASTIX multilayer mirror with point focus, and diffraction patterns were recorded with an Eiger R 1M 2D detector (Dectris, Baden-Daettwill, Switzerland). Microparticles (EA-InSc, EA-InA, GA-InSc and GA-InA) were loaded into 1.0 mm diameter glass capillaries (Charles Supper Company, EE. UU.), flame-sealed, and exposed at 25 °C for 900 s. A single frame was collected at a sample-to-detector distance of 115 mm and calibrated using a LaB6 standard. The resulting patterns were processed using SAXSDrive software v2.02.295.15850 (Anton Paar, Graz, Austria), including transmission and geometry correction, background subtraction, and reduction to 1D patterns (scattering intensity vs. 2θ).

For crystallinity index (CI) determination, the angular range of 5–35° in 2θ was analyzed. Each sample pattern was corrected by subtracting a previously obtained 100% amorphous reference pattern. The remaining signal corresponded to the crystalline fraction, and its area was divided by the total area of the sample pattern to calculate the crystalline proportion, according to Equation (2).

(2) $$CI\left(\%\right)=\frac{Integrate\;intensity\;of\;crystalline\;peaks}{Total\;integrate\;intensity\;of\;scattering\;}\times 100$$

#### 2.2.2. Encapsulation Efficiency (EE)

Total EA and GA: Microparticles (100 mg) of each system were dispersed in 4 mL of methanol, vortexed for 1 min (FineVortex, FinePCR, Gunpo-si Republic of Korea), sonicated for 5 min (Elmasonic E 30 H, Elma-Hans Schmidbauer GmbH, Singen, Germany), and allowed to stand for 5 min. This procedure was repeated twice. The mixture was then centrifuged (Hettich Universal 329R, Tuttlingen, Germany) at 7690× *g* for 10 min at 4 °C (Universal 320 R, Hettich, Germany), and the supernatant was collected in a 10 mL volumetric flask. The resulting pellet was resuspended in 1 mL of distilled water (75 °C): methanol (1:3 *v*/*v*), and the same extraction procedure was repeated. The supernatants from EA microparticles were combined, brought to 10 mL with methanol and analyzed by HPLC. For GA microparticles, the combined supernatants were subjected to solvent evaporation using a rotary evaporator (R-100, Büchi, Switzerland), and the residue was redissolved in acetonitrile:water (1:1 *v*/*v*) before HPLC analysis. Surface EA or GA: Microparticles (EA-InSc, EA-InA, GA-InSc and GA-InA, 100 mg) were dispersed in 4 mL of ethanol:ethyl acetate (1:2 *v*/*v*) and gently stirred for 1 min. An aliquot (2 mL) was centrifuged at 3900× *g* for 3 min. The supernatant was transferred to a 10 mL volumetric flask and adjusted to volume with ethanol:ethyl acetate. The solvent was then evaporated under a nitrogen stream. The residue of EA samples was resuspended in methanol and injected into the HPLC, whereas the residue from GA samples was resuspended in acetonitrile:water (1:1) before HPLC analysis.

EA and GA chromatographic analysis: Quantification of EA and GA was performed using an HPLC system (Alliance e2695, Waters, Milford, MA, USA) equipped with a photodiode array detector (Waters 2998, Waters, USA) and a C18 column (5 μm, 4.6 mm i.d. × 250 mm; Symmetry, Waters, Dublin, Ireland), following the methods described by De Cristo Soares et al. [2] for GA and Li et al. [24] for EA. Quantification was performed using external calibration curves: 1–50 μg/mL for EA (R^2^ = 1.00) and 1–100 μg/mL for GA (R^2^ = 0.99).

The encapsulation efficiency (EE) of EA and GA was calculated according to Equation (3).

(3) $$EE\;\left(\%\right)=\frac{Total\;EA\;or\;GA-Surface\;EA\;or\;GA}{Total\;EA\;or\;GA}\times 100$$

#### 2.2.3. Particle Size and Morphology

Microparticle size and size distribution for EA-InSc, EA-InA, GA-InSc and GA-InA were determined by light scattering using a particle size analyzer (Partica LA-960, Horiba, Japan) and reported as the volume-weighted mean diameter (D_4,3_) [16]. Measurements were performed in dry mode using a PowerderJet accessory with compressed air at 0.3 MPa.

The morphology and surface structure of the microparticles (EA-InSc, EA-InA, GA-InSc and GA-InA) were examined using high-resolution scanning electron microscopy (SEM; FE-SEM, Inspect-F50, FEI, Eindhoven, Netherlands) equipped with a secondary electron detector (SED), operating at 2 kV. Prior to imaging, the microparticles were coated with a thin gold layer (10 nm) using a sputter coater (Cressington 108, Ted Pella Inc., California, USA) equipped with a thickness controller (Cressington MTM-20). Micrographs were captured and analyzed with EDS 7424 software (Oxford Instruments, Oxford, UK).

#### 2.2.4. Spray-Drying Yield

Yield of the process was calculated according to Equation (4).

(4) $$Yield\;\left(\%\right)=\frac{Microparticles\;obtained\;after\;spray\;drying\;\left(g\right)}{Solid\;content\;in\;infeed\;spray\;drying\;\left(g\right)}\times 100$$

### 2.3. In Vitro Simulated Gastrointestinal Digestion

#### 2.3.1. Formulation of Model Food Matrices with Ellagic Acid- and Gallic Acid-Inulin Microparticles

Microparticles (EA-InSc, EA-InA, GA-InSc and GA-InA 0.2 g) were incorporated into four model food matrices (4.8 g): a carbohydrate (sucrose 6% + pectin 2%), a protein matrix (whey protein concentrate 3.1%), a lipid matrix (sunflower oil-in-water emulsion, 1.8%), and a complete matrix combining sucrose, pectin, whey protein concentrate, and sunflower oil-in-water emulsion. Water was used as the control matrix.

#### 2.3.2. Preparation of Model Food Matrices

Carbohydrate matrix (C): 0.3 g of sucrose and 0.1 g of pectin were dispersed in distilled water under constant stirring for 30 min at 20 °C. The mixture was then heated to 37 °C, and 0.2 g of microparticles containing either EA or GA with semicrystalline or amorphous inulin were incorporated (C+EA-InSc, C+GA-InSc, C+EA-InA, and C+GA-InA).

Protein matrix (P): 0.2 g of whey protein concentrate was dispersed in distilled water under constant stirring for 30 min at 20 °C. The mixture was subsequently heated to 37 °C, and 0.2 g of microparticles containing either EA or GA with semicrystalline or amorphous inulin were incorporated (P+EA-InSc, P+GA-InSc, P+EA-InA, and P+GA-InA).

Lipid matrix (L): 0.1 g of sunflower oil was homogenized with soy lecithin and distilled water at 20,000 rpm for 2 min using a homogenizer (Polytron PT-2100, Kinematica AG, Malters, Switzerland). The resulting emulsion was heated to 37 °C, and 0.2 g of microparticles containing either EA or GA with semicrystalline or amorphous inulin were incorporated (L+EA-InSc, L+GA-InSc, L+EA-InA, and L+GA-InA).

Blend matrix (B): all components used in the previous preparations (carbohydrate, protein and lipid) were combined, and 0.2 g of microparticles containing either EA or GA with semicrystalline or amorphous inulin were incorporated (B+EA-InSc, B+GA-InSc, B+EA-InA, and B+GA-InA).

#### 2.3.3. In Vitro Simulated Digestion for the Microparticles and Model Food Matrices

A sample of 0.2 g of EA-InSc, EA-InA, GA-InSc, or GA-InA (containing 1.9 mg of EA or GA) was either dispersed in water to a final mass of 5 g or incorporated into each model food matrix (4.8 g) to yield a total of 5 g. Both the microparticles alone and those incorporated into food matrices were subjected to simulated in vitro digestion in triplicate, following the INFOGEST protocol [22], using a 50 mL jacketed beaker connected to a recirculating bath (refrigerated JSRC-13C, JS Research Inc., Gongju, Republic of Korea) to maintain 37 °C, while stirring at 250 rpm with a magnetic stirrer (Heidolph Instruments, Schwabach, Germany). Simulated digestive fluids for the oral, gastric, and intestinal stages were prepared according to the INFOGEST protocol [22]. At the end of each digestion stage, aliquots of 1 mL (oral phase), 2 mL (gastric phase), and 5 mL (intestinal phase) were collected, centrifuged at 3900× *g* for 10 min at 5 °C, and the resulting supernatants (0.75 mL, 1.50 mL and 3.75 mL for oral, gastric and intestinal phases, respectively) were stored at −20 °C for further analysis.

#### 2.3.4. Quantification of Ellagic Acid or Gallic Acid Released During In Vitro Simulated Digestion

To quantify the EA or GA released from the microparticles (EA-InSc, EA-InA, GA-InSc and GA-InA) in each digestion stage, the supernatants were thawed, acidified with formic acid to pH 2, and diethyl ether was added at a 1:1 (*v*/*v*) ratio. The mixtures were vortexed for 1 min and incubated in an orbital shaker (JSSI-100C, JS Research, Gongju, Republic of Korea) at 140 rpm and 10 °C for 14 h. The organic phase (diethyl ether containing EA or GA) was collected in a test tube. This extraction was repeated twice (4 h each), and the organic phase was pooled. The solvent was evaporated, and the extracted polyphenols were resuspended in methanol (for EA) or in acetonitrile:water (1:1, *v*/*v*) (for GA). The samples were filtered through a 13 mm PTFE syringe filter with a 0.22 µm pore size (Macherey-Nagel GmbH, Düren, Germany). Quantification of EA and GA was performed by HPLC as detailed in Section 2.2.2.

#### 2.3.5. Bioaccessibility of Ellagic Acid or Gallic Acid

Bioaccessibility is defined as the fraction of a compound that remains soluble and available for absorption in the intestine after digestion. The bioaccessibility of EA and GA was calculated as the ratio between their concentration in the soluble fraction of the intestinal phase and their corresponding initial concentration, according to Equation (5).

(5) $$Bioaccesibility\;\left(\%\right)=\frac{EA\;or\;GA\;content\;\left(mg\right)\;in\;intestinal\;phase}{Initial\;EA\;or\;GA\;content\;\left(mg\right)}\;$$

#### 2.3.6. Determination of Antioxidant Activity

The antioxidant activity (AA) of EA and GA released from microparticles (EA-InSc, EA-InA, GA-InSc, GA-InA) during the different stages of simulated gastrointestinal digestion was determined using three methods: the DPPH radical scavenging assay, the ABTS^•+^ decolorization assay, and the modified ferricyanide (Fe^3+^-reducing power) method. Trolox calibration curves were prepared with different concentration ranges, which were adjusted to match the levels of GA and EA present in each digestive phase in order to ensure accurate determination of antioxidant activity.

DPPH assay: the assay was performed as described by Odriozola-Serrano et al. [25]. Trolox calibration curves were prepared in the range of 12.5–200 ppm for GA and 2.5–100 ppm for EA. A specific calibration curve was prepared for each digestion phase (oral, gastric and intestinal), using the corresponding blank solutions containing empty microparticles (without polyphenols) subjected to identical digestion conditions (pH and enzymes). All calibration curves showed an R^2^ ≥ 0.99.

ABTS assay: The ABTS method was performed as described by Re et al. [26]. Trolox calibration curves were prepared in the range of 0.1–1.2 mM for GA and 10–800 µM for EA. Phase-specific calibration curves (oral, gastric, intestinal) were generated using corresponding blanks, with R^2^ ≥ 0.99 in all cases. For analysis, 980 µL of ABTS^•+^ solution was mixed with 20 µL of sample, incubated for 10 min at 25 °C, and absorbance was measured at 734 nm.

Modified ferricyanide assay: The method (with incubation) was performed as described by Berker et al. [27]. Trolox calibration curves were prepared in the range of 0.05–0.7 mM for GA and 5–400 µM for EA, with phase-specific blanks. All calibration curves showed R^2^ ≥ 0.99.

## 3. Results and Discussion

### 3.1. Experimental Design for Encapsulation of Ellagic Acid

A composite central experimental design (4 experimental points, 4 axial points, and 4 central points) was applied to evaluate the effect of ethanol concentration and T_e_ on the EE of EA and CI (Table 1).

The CI of EA–inulin microparticles ranged from 1.0% to 24.6% (Table 1). These values were lower than those reported for epicatechin- and quercetin-inulin microparticles, with CI values of 61.2% and 60.0% at an inlet temperature of 15 °C, and 41.6% and 51.1% at 90 °C, respectively [16]. Comparable CI values have also been observed in microparticles containing other phenolic compounds, such as curcumin and capsaicin with high-amylose corn starch (14.61% and 14.65%, respectively [28]), and in black tea extract microparticles formulated with pectin, sodium caseinate, and pectin+sodium caseinate (5.75%, 6.17%, and 4.8%, respectively; [29]).

**Figure 1:**
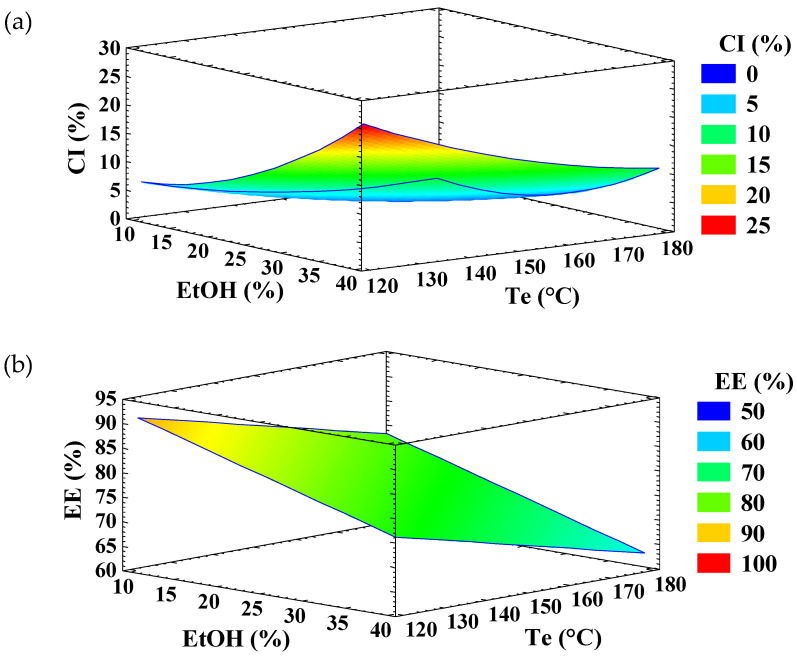
Response surface graph for the variable (**a**) crystallinity index (CI) and (**b**) encapsulation efficiency (EE) of ellagic acid (EA)—inulin microparticles.

According to the analysis of variance (Table 1), the linear and quadratic terms of EtOH percentage and T_e_, as well as their interaction, were statistically significant (*p* < 0.05) for CI. The experimental data fitted the model well, as indicated by a high adjusted coefficient of determination (R^2^ adj = 99.0), residual values under 6.0, and a non-significant lack-of-fit. The response surface graph (Figure 1a) shows that CI increased with ethanol concentration, which can be attributed to the role of ethanol as an antisolvent. Ethanol promotes the formation of inulin crystallites within the infeed dispersion, thereby increasing the CI of EA microparticles. Similar effects of ethanol have been reported for lactose [21], cyclodextrines [30], and amylose [31]. Other solvents, such as methanol, acetone, and n-propanol, have also been described as antisolvents [32]. In contrast, increasing the T_e_ slightly increased the CI of the microparticles (Figure 1a), particularly at low ethanol concentrations.

The EE of EA ranged from 57.6% to 87.5% (Table 1). Comparable EE values have been reported for semicrystalline epicatechin-inulin and quercetin-inulin microparticles (68.8 and 67.8%, respectively), whereas their amorphous microparticles showed lower EE values (41.6% and 51.1%, respectively). In studies where crystallinity was not determined, lower EE values were reported, such as in quercetin-inulin, naringenin-inulin and epicatequin-inulin microparticles (39.7–73.3%, 30.4–50.5% and 50.1–85.2%, respectively) [33]; and quercetin- or epicatechin-(inulin+soy protein isolate) microparticles (26.7–54.1% and 36.7–77.0%, respectively) [34]. These findings suggest that the EE of phenolic compounds may vary depending on their structural characteristics and the physical state of the microparticles.

Only the linear term of ethanol concentration and the T_e_ showed a significant influence (*p* < 0.05) on the EE of EA (Table 1). The experimental data fitted the model adequately, with an adjusted R^2^ of 74.5% and a non-significant lack-of-fit. The response surface graph (Figure 1b) showed that the EE of EA increased as ethanol concentration and T_e_ decreased. The reduction in EE with increasing ethanol concentration may be attributed to molecular competition for hydrogen bonding sites: the hydroxyl groups of ethanol may interact preferentially with the hydroxyl and/or carbonyl groups of EA, limiting its ability to form hydrogen bonds with inulin.

EA–inulin microparticles were successfully obtained in two distinct physical states—amorphous (EA-InA) and semicrystalline (EA-InSc)—through multiple-response optimization using the desirability function. For the semicrystalline formulation, both CI and EE were simultaneously maximized, with desirability of 0.85 and optimal conditions predicted at 36.5% ethanol and a T_e_ of 114 °C (Table 2). In contrast, the amorphous formulation was obtained by simultaneously minimizing both parameters (desirability of 0.98), with optimal conditions of 6.8% ethanol and an inlet air temperature of 148 °C (Table 2). Using these optimized conditions, GA–inulin microparticles were also obtained in semicrystalline (GA-InSc) and amorphous (GA-InA) states.

### 3.2. Characterization of EA- and GA-Inulin Microparticles Obtained Under Optimal Conditions

#### 3.2.1. Crystallinity Index (CI)

**Figure 2:**
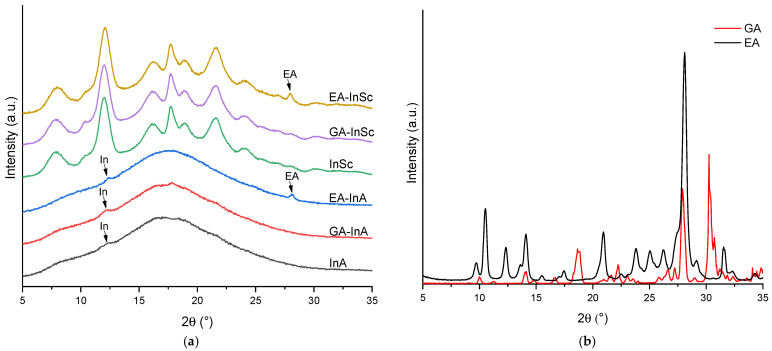
X-ray diffraction patterns of (**a**) amorphous and semicrystalline ellagic acid (EA), gallic acid (GA) and empty inulin microparticles, and (**b**) EA and GA.

Figure 2a shows the X-ray diffraction (XRD) patterns of EA- and GA-inulin microparticles in the 2θ range of 5–35°. Both GA-InA and EA-InA systems exhibited a broad diffuse halo (Figure 2a), characteristic of an amorphous state. In addition, small peaks at 12° were observed in both amorphous systems, attributed to crystalline domains of inulin. In EA-InA, a further peak at 28° was also detected, suggesting the presence of residual EA crystalline domains (Figure 2b).

The semicrystalline systems (GA-InSc and EA-InSc) showed sharp diffraction peaks superimposed on a broad halo, consistent with a semicrystalline structure (Figure 2a). In EA-InSc, the peak at 28° was attributed to EA crystalline domains. In addition, the absence of a peak at 10.6° indicated that both semicrystalline EA- and GA-inulin were in their monohydrated form [35].

The CI for EA-InA y GA-InA was 2.1% and 1.7%, respectively, whereas higher CI values of 23.5% and 20.0% were found for EA-InSc and GA-InSc (Table 2). The comparable CI values of both semicrystalline systems suggest that the biopolymer is the main factor influencing crystallinity. In contrast, Morelo et al. [16] reported much higher values of 60% for semicrystalline quercetin–inulin and 61.2% for semicrystalline epicatechin–inulin microparticles, whereas amorphous quercetin– and epicatechin–inulin microparticles exhibited much lower values, ranging from 1.73% to 2.30%. These findings indicate that the infeed dispersion formulation, spray-drying operating conditions, and the type of phenolic compound strongly influence the CI of the resulting microparticles. These findings suggest that the crystallinity index of the resulting microparticles is influenced by the infeed dispersion formulation, the spray-drying operating conditions, and the specific type of phenolic compound used.

#### 3.2.2. Encapsulation Efficiency (EE)

Encapsulation efficiency represents the interaction between phenolic compounds with inulin, mainly through hydrogen bonding. The EE of GA microparticles was 99.0% for GA-InSc and 99.2% for GA-InA (Table 2). These values were higher than those reported for GA-In microparticles in other studies, which reached 83% [36] and 81% [37], although crystallinity was not assessed. In other studies, GA was encapsulated with modified potato starch with EE values of 70–84% [7], with pectin and alginate 79–90% [8], and with whey protein concentrate and Ulmus davidiana polysaccharides 79–83% [6].

The EE of EA microparticles was 82.6% for EA-InSc and 83.4% for GA-InA (Table 2), which was lower than that of GA-In microparticles. The values obtained here were, however, higher than those reported for EA-chitosan microparticles, with EE values of 50% [11], and for EA-chitosan and EA-chitosan/Tween-80 nanoparticles, with EE values of 71.3% and 79.4%, respectively [12].

In this study, the EE of GA-In and EA-In was not influenced significantly by the CI. Differences between the present results and those previously reported may be attributed to the nature of the phenolic compound, the structural characteristics of the biopolymer, as well as formulation and processing conditions. The higher EE for GA can be explained by its molecular characteristics. GA is a small molecule that presents three hydroxyl groups, which facilitate its water solubility and interaction with inulin by hydrogen bonding during spray drying. In contrast, EA has a more complex structure and a rigid planar structure with poor water solubility. These structural characteristics limit its interaction with inulin, thereby resulting in lower EE. Therefore, the higher EE observed for GA compared with EA can be attributed to differences in molecular size, solubility, and polarity, which influence their interaction with inulin.

#### 3.2.3. Particle Size and Morphology

The microparticle size ranged from 3.2 to 3.8 µm (Table 2), with no significant differences attributable to the type of polyphenol or to the physical state of the microparticles. Larger particle sizes have been reported in GA-In microparticles (4.4 µm, [37]), and in epicatechin and quercetin-inulin microparticles (4.4–6.1 µm, [16]). These variations among studies may be attributed to differences in infeed formulation and drying conditions.

**Figure 3:**
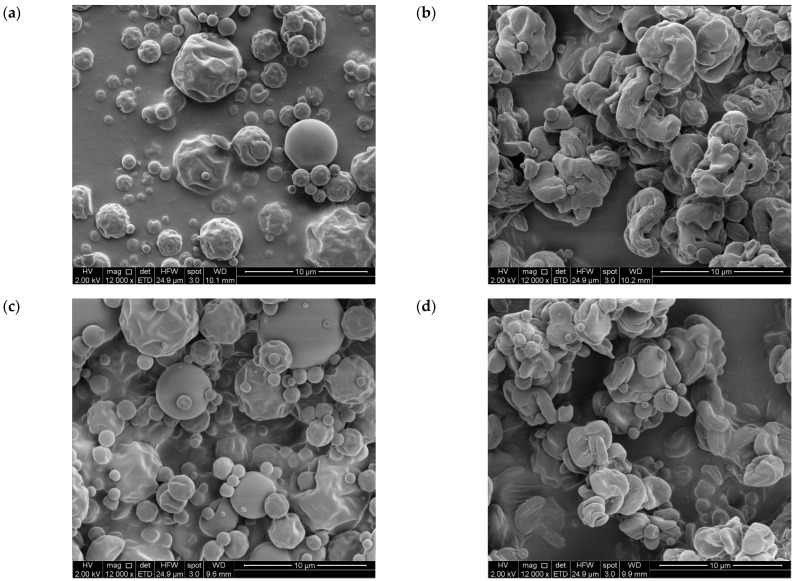
SEM micrographs of amorphous and semicrystalline gallic acid- and ellagic acid-inulin microparticles (**a**) GA-InA, (**b**) GA-InSc, (**c**) EA-InA and (**d**) EA-InSc (magnification: 12,000).

Figure 3 shows the SEM images of GA microparticles (GA-InA, GA-InSc, Figure 3a,b respectively) and EA microparticles (EA-InA, EA-InSc, Figure 3c,d respectively). The amorphous systems (EA-InA and GA-InA) exhibited a spherical shape with smooth or slightly rough surfaces, with some irregular particles showing dents. In contrast, the semicrystalline systems (EA-InSc and GA-InSc) displayed more irregular morphologies with markedly rougher, spiral-like surfaces. The observed morphological differences may be attributed to factors such as drying process conditions (inlet temperature and infeed temperature) and infeed formulation. These parameters have been reported to directly affect droplet size, viscosity, and particle cohesion, which in turn may influence surface roughness, porosity, and the physical state (amorphous/crystalline) of the microparticles [38]. However, these aspects were not measured in this study and therefore cannot be asserted.

The microparticle yield ranged from 91.6 to 94.5, indicating low adhesion of the powders to the walls of the drying chamber. A slightly higher yield was obtained for semicrystalline microparticles (EA-InSc and GA-InSc) than for the amorphous microparticles, which can be attributed to the lower stickiness of the semicrystalline structures during spray drying. This is due to the semicrystalline inulin having ordered regions that reduce molecular mobility and water uptake.

### 3.3. In Vitro Simulated Digestion

#### 3.3.1. Release Profile of GA and EA

**Figure 4:**
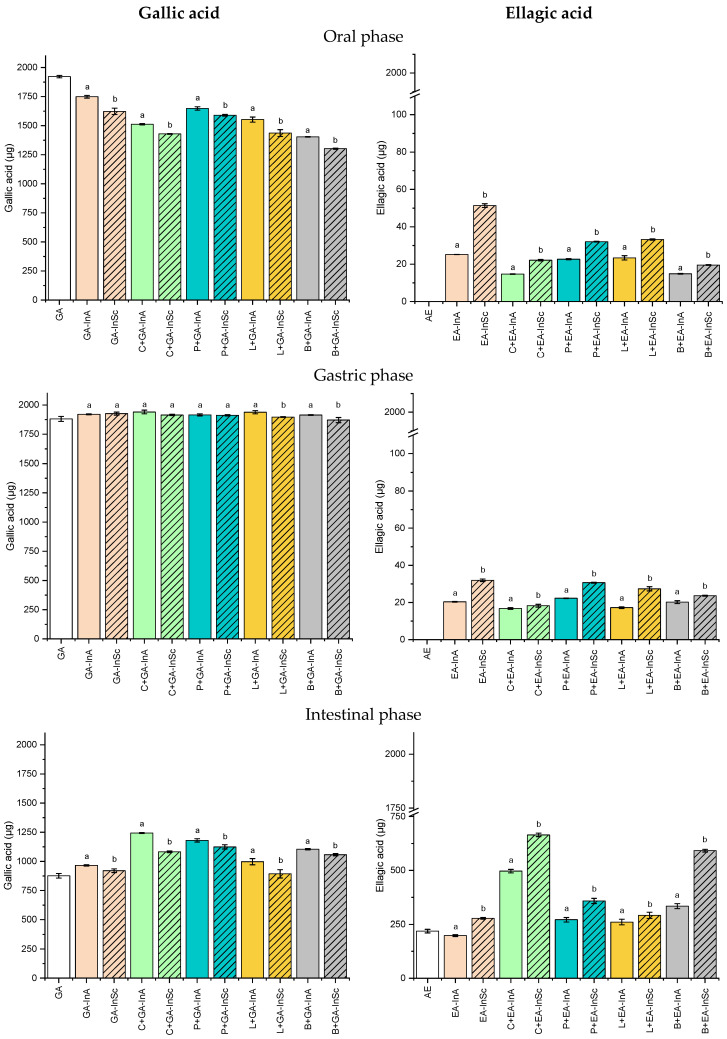
Soluble gallic acid (GA) and ellagic acid (EA) content in the oral, gastric, and intestinal phase for amorphous (InA) and semicrystalline (InSc) microparticles, either without or with microparticles incorporated into food matrices during the simulated in vitro digestion. C: carbohydrate matrix; P: protein matrix; L: lipid matrix; B: blend matrix. Different lowercase letters indicate significant differences (*p* ≤ 0.05) between semicrystalline and amorphous microparticle systems with the same polyphenol and food matrix.

Figure 4 shows the release profile of GA and EA from microparticles (GA-InA, GA-InSc, EA-InA and EA-InSc) either without or with microparticles incorporated into food matrices during the simulated in vitro gastrointestinal digestion.

In the oral phase, GA release was significantly higher from amorphous microparticles (GA-InA, 1749 µg) than semicrystalline microparticles (GA-InSc, 1623 µg). The same trend was found when GA-microparticles were incorporated into food formulations. GA release from GA-InA and GA-InSc incorporated into food formulations, ranging from 1404 to 1648 µg and 1303 to 1590 µg, respectively, indicating that the amorphous physical state facilitates GA mobility and release compared to the semicrystalline state, due to its disordered molecular chains [39]. However, the blend systems containing both GA-InA and GA-InSc exhibited the lowest GA release, suggesting that in these systems the network structure of the food matrix decreased GA diffusion during the oral digestion phase.

The release of GA was near 100% in gastric digestion for GA-InA and GA-InSc, both in the absence and presence of food matrices, indicating that acidic conditions prevented GA degradation similar to rutin, caffeic acid and rosmarinic acid [40,41]. In contrast, lower GA contents were detected during the intestinal phase in GA-InA, GA-InSc, and in food matrices containing GA-microparticles. This reduction may result from GA degradation under alkaline conditions [41], its binding to pancreatic enzymes through hydrogen bonding and hydrophobic interactions [42], complexation with bile salts, or interactions with food matrix components [43]. The bioaccessibility of GA was approximately 50% in GA-InA and GA-InSc (Figure 4, intestinal phase).

The incorporation of GA microparticles into food matrices increased GA bioaccessibility in carbohydrate-, protein-, and blend-containing systems, particularly for GA-InA microparticles (57.8–65.1%). These bioaccessibility values are higher than those previously reported for GA in other studies. For instance, Ydjedd et al. [44] found that, in phenolic compounds from carob pulp (*Ceratonia siliqua* L.) encapsulated by spray drying, GA bioaccessibility was only 1.4%. Similarly, López-Astorga et al. [1] did not detect GA in the intestinal phase during the digestion of grape pomace polyphenolic extracts microencapsulated with maltodextrin and gum arabic by spray drying, suggesting complete degradation of GA at this stage.

Contrary to GA, the release of EA during the oral phase was low, with values below 51.4 µg (2.7% release), corresponding mainly to EA located on the microparticle surface. No significant release was detected in the gastric phase, where EA content remained constant in both free microparticles and microparticles incorporated into food matrices. This limited release can be attributed to the low aqueous solubility of EA, since this compound is not ionized at gastric conditions (pH of 2.0). However, a higher release occurred in the intestinal phase, where semicrystalline microparticles (EA-InSc) exhibited significantly greater EA release (277.5 µg) than their amorphous counterparts (EA-InA, 198.2 µg). This behavior may be due to an increase in EA solubility in aqueous media under alkaline conditions by deprotonation of hydroxyl groups [45]. In the amorphous state, characterized by highly disordered chains, EA can establish more numerous interactions with inulin by hydrogen bonding [46], thereby hindering its release into the intestinal medium.

With respect to the effect of the food matrix, it was observed that carbohydrate-based systems exhibited the highest EA release (34.2% for EA-InSc and 25.9% for EA-InA) compared with the other systems studied. Such behavior is likely related to the presence of pectin, which forms a three-dimensional network in solution, preventing EA precipitation in the intestinal medium [47]. EA bioaccessibility was significantly higher for semicrystalline microparticles (EA-InSc, 14.3%) than for the amorphous system (EA-InA, 10.3%) (Figure 4, intestinal phase). When incorporated into food matrices, EA exhibited increased bioaccessibility in both carbohydrate and blend-based systems, with semicrystalline microparticles (EA-InSc), showing the highest values (34.2% and 30.5%, respectively).

This EA profile suggests that encapsulation may protect the EA from premature degradation in the upper gastrointestinal tract, allowing its targeted release in the intestine. However, the static nature of the INFOGEST method represents a limitation, since it does not allow a dynamic balance between the absorbed and released EA. Although ellagic acid does not appear to be fully released during the intestinal phase, the fraction that reaches the colon may undergo microbial metabolism into urolithins, bioactive metabolites that can contribute to overall health effects [45].

#### 3.3.2. Effect of In Vitro Simulated Digestion on Antioxidant Activity

The AA during simulated in vitro digestion was evaluated using DPPH (2,2-diphenyl-1-picrylhydrazyl), ABTS (2,2-azinobis-3-ethylbenzothiazoline-6-sulphonic acid), and modified ferricyanide (Fe^3+^-reducing power) assays. These methods were chosen because they rely on different antioxidant mechanisms (mainly, hydrogen atom transfer and/or electron transfer), and the response can vary depending on the structural features of the individual phenolic compounds. Antioxidant activity was determined in each phase of the gastrointestinal digestion. Table 3 and Table 4 show the evolution of AA during simulated in vitro digestion of GA- and EA-microparticles, respectively, both in the absence and presence of food matrices.

In the oral phase, GA-InA exhibited slightly higher AA values than GA-InSc, both as free microparticles and when incorporated into food matrices (Table 3). This effect was attributed to the greater surface area of the amorphous microparticles, which facilitated GA release. During the gastric phase, AA values obtained from DPPH, ABTS and ferricyanide assays were also higher for GA-InA. The highest AA was observed at this stage, coinciding with the maximum GA release and consistent with previous studies [48]. While DPPH values were comparable to those in the oral phase, ABTS and ferricyanide assays showed enhanced AA in food matrices containing proteins or protein–carbohydrate blends. This increase may be related to pepsin hydrolysis of proteins, generating peptides and amino acids with antioxidant activity [49]. A subsequent decrease in GA content during intestinal digestion (Figure 4) was consistent with the reduction in AA detected by all three assays, which can be explained by partial GA degradation at neutral pH (7.0) and/or its interaction with intestinal-phase components or food matrix constituents as described above.

The evolution of AA, in EA-microparticles (Table 4), whether free or embedded in food matrices, showed that semicrystalline microparticles (EA-InSc) displayed higher AA values than amorphous microparticles (EA-InA) in all digestion phases, consistent with the EA release pattern observed during simulated digestion (Figure 4). This trend contrasted with GA-microparticles (Table 3), where amorphous systems showed higher AA than semicrystalline ones. The distinct evolution of AA between EA- and GA-microparticles can be attributed to their structural and physicochemical differences, as was previously explained.

In both the oral and gastric phases, AA values were low for EA-free microparticles as well as those embedded in food matrices. The low AA in the gastric phase was consistent with the limited EA release under these conditions, which can be attributed to its poor solubility in acidic aqueous environments where EA remains predominantly protonated.

In contrast, a marked increase in AA (DPPH, ABTS, ferricyanide) was detected in the intestinal phase. At this stage, the higher pH promotes EA deprotonation, improving its solubility and thereby enhancing its release [45]. Among the assays, ferricyanide recorded the highest AA values, reflecting the fact that the predominant antioxidant mechanism of EA in polar environments is electron transfer [50].

**Table 3:** Effect of the food matrix on the antioxidant activity of gallic acid (GA) released from semicrystalline (GA-InSc) and amorphous (GA-InA) microparticles during simulated in vitro gastrointestinal digestion.

	Oral Phase (µmol Trolox/g Microparticles)			Gastric Phase (µmol Trolox/g Microparticles)			Intestinal Phase (µmol Trolox/g Microparticles)		
	DPPH	ABTS	Ferricyanide	DPPH	ABTS	Ferricyanide	DPPH	ABTS	Ferricyanide
Semicrystalline microparticles									
GA-InSc	23.7 ± 0.7^b^	26.3 ± 0.1 ^a^	17.5 ± 0.1 ^b^	24.4 ± 0.2 ^a^	30.9 ± 0.2 ^cd^	18.3 ± 0.1 ^d^	16.4 ± 0.1 ^b^	11.4 ± 0.1 ^d^	10.2 ± 0.3 ^b^
C+GA-InSc	22.9 ± 0.2 ^bc^	24.4 ± 0.3 ^b^	17.7 ± 0.1 ^b^	23.7 ± 0.5 ^a^	30.4 ± 0.3 ^d^	18.5 ± 0.1 ^d^	16.6 ± 0.4 ^b^	11.9 ± 0.0 ^c^	9.6 ± 0.2 ^b^
P+GA-InSc	26.8 ± 0.2 ^a^	26.6 ± 0.2 ^a^	19.5 ± 0.1 ^a^	24.0 ± 0.5 ^a^	51.9 ± 0.3 ^a^	34.7 ± 0.7 ^a^	18.1 ± 0.2 ^a^	14.5 ± 0.0 ^a^	12.4 ± 0.1 ^a^
L+GA-InSc	22.1 ± 0.4 ^c^	24.5 ± 0.2 ^b^	15.5 ± 0.1 ^c^	21.1 ± 0.2 ^b^	31.6 ± 0.3 ^c^	19.9 ± 0.0 ^c^	12.9 ± 0.2 ^c^	11.8 ± 0.0 ^c^	5.9 ± 0.3 ^c^
B+GA-InSc	15.6 ± 0.1 ^d^	18.6 ± 0.2 ^c^	13.1 ± 0.3 ^d^	20 ± 0.5 ^b^	39.1 ± 0.2 ^b^	33.2 ± 0.1 ^b^	14.2 ± 0.1 ^d^	13.8 ± 0.0 ^b^	10.2 ± 0.3 ^b^
Amorphous microparticles									
GA-InA	27.0 ± 0.3 ^a^	28.4 ± 0.3 ^b^	18.6 ± 0.1 ^b^	25.7 ± 0.2 ^a^	34.7 ± 0.2 ^c^	19.9 ± 0.2 ^d^	18.2 ± 0.2 ^c^	12.4 ± 0.0 ^c^	11.0 ± 0.5 ^c^
C+GA-InA	25.5 ± 0.0 ^b^	26.2 ± 0.2 ^c^	18.8 ± 0.0 ^b^	25.7 ± 0.2 ^a^	33.5 ± 0.0 ^d^	21.5 ± 0.4 ^c^	18.7 ± 0.0 ^b^	13.6 ± 0.2 ^b^	10.7 ± 0.2 ^c^
P+GA-InA	27.6 ± 0.1 ^a^	28.8 ± 0.2 ^a^	20.4 ± 0.2 ^a^	25.4 ± 0.1 ^a^	53.8 ± 0.6 ^a^	36.6 ± 0.1 ^a^	19.3 ± 0.1 ^a^	14.9 ± 0.2 ^a^	13.4 ± 0.1 ^a^
L+GA-InA	24.5 ± 0.4 ^c^	26.5 ± 0.0 ^c^	16.2 ± 0.0 ^c^	23.3 ± 0.2 ^b^	35.4 ± 0.2 ^c^	22.3 ± 0.6 ^c^	13.5 ± 0.1 ^d^	12.5 ± 0.4 ^c^	9.3 ± 0.3 ^d^
B+GA-InA	19.2 ± 0.2 ^d^	21.3 ± 0.1 ^d^	15.1 ± 0.2 ^d^	22.7 ± 0.1 ^c^	50.3 ± 0.3 ^b^	34.7 ± 0.3 ^b^	15.0 ± 0.2 ^e^	15.0 ± 0.1 ^a^	12.1 ± 0.4 ^b^

GA: Gallic acid; GA-InSc: semicrystalline inulin–GA microparticles; GA-InA: amorphous inulin–GA microparticles; C: carbohydrate matrix (sucrose + pectin); P: protein matrix (whey protein concentrate); L: lipid matrix (sunflower oil); B: blend matrix. Results are expressed as mean ± standard deviation of three experiments. Lowercase letters indicate statistical differences (*p* ≤ 0.05) among systems in each digestion phase within each physical state of the microparticles (semicrystalline and amorphous) for each antioxidant activity assay.

**Table 4:** Effect of the food matrix on the antioxidant activity of ellagic acid (EA) released from semicrystalline (EA-InSc) and amorphous (EA-InA) microparticles during simulated in vitro gastrointestinal digestion.

	Oral Phase (µmol Trolox/g Microparticles)			Gastric Phase (µmol Trolox/g Microparticles)			Intestinal Phase (µmol Trolox/g Microparticles)		
	DPPH	ABTS	Ferricyanide	DPPH	ABTS	Ferricyanide	DPPH	ABTS	Ferricyanide
Semicrystalline microparticles									
EA-InSc	0.7 ± 0.04 ^bc^	1.0 ± 0.03 ^bc^	0.6 ± 0.02 ^a^	0.4 ± 0.03 ^c^	0.4 ± 0.04 ^bc^	0.3 ± 0.01 ^d^	1.5 ± 0.03 ^c^	1.8 ± 0.02 ^c^	2.3 ± 0.12 ^d^
C+EA-InSc	0.6 ± 0.01 ^c^	0.7 ± 0.01 ^d^	0.4 ± 0.03 ^c^	0.5 ± 0.05 ^bc^	0.3 ± 0.02 ^c^	0.4 ± 0.01 ^c^	3.3 ± 0.05 ^a^	3.6 ± 0.14 ^a^	6.5 ± 0.22 ^b^
P+EA-InSc	0.6 ± 0.01 ^c^	1.1 ± 0.06 ^b^	0.5 ± 0.01 ^b^	0.5 ± 0.02 ^bc^	0.5 ± 0.01 ^b^	0.3 ± 0.02 ^d^	1.1 ± 0.05 ^d^	2.1 ± 0.05 ^b^	3.6 ± 0.20 ^c^
L+EA-InSc	0.7 ± 0.00 ^b^	0.9 ± 0.02 ^cd^	0.5 ± 0.01 ^b^	0.8 ± 0.05 ^a^	0.7 ± 0.04 ^a^	0.7 ± 0.02 ^a^	1.1 ± 0.01 ^d^	1.0 ± 0.01 ^d^	1.8 ± 0.14 ^e^
B+EA-InSc	0.8 ± 0.02 ^a^	1.4 ± 0.04 ^a^	0.5 ± 0.03 ^b^	0.5 ± 0.06 ^b^	0.5 ± 0.02 ^b^	0.6 ± 0.01 ^b^	2.2 ± 0.1 ^b^	3.5 ± 0.02 ^a^	7.0 ± 0.05 ^a^
Amorphous microparticles									
EA-InA	0.5 ± 0.00 ^b^	0.8 ± 0.03 ^b^	0.2 ± 0.01 ^c^	0.3 ± 0.00 ^c^	0.3 ± 0.01 ^b^	0.1 ± 0.03 ^c^	1.1 ± 0.07 ^c^	1.4 ± 0.08 ^d^	1.6 ± 0.06 ^d^
C+EA-InA	0.5 ± 0.03 ^b^	0.7 ± 0.01 ^c^	0.3 ± 0.02 ^ab^	0.4 ± 0.05 ^ab^	0.2 ± 0.02 ^b^	0.3 ± 0.01 ^b^	3.1 ± 0.07 ^a^	2.5 ± 0.08 ^b^	5.4 ± 0.08 ^b^
P+EA-InA	0.6 ± 0.01 ^a^	0.6 ± 0.01 ^d^	0.4 ± 0.02 ^a^	0.4 ± 0.01 ^ab^	0.2 ± 0.01 ^b^	0.1 ± 0.01 ^c^	1.0 ± 0.03 ^c^	1.8 ± 0.07 ^c^	2.7 ± 0.16 ^c^
L+EA-InA	0.5 ± 0.01 ^b^	0.7 ± 0.01 ^c^	0.3 ± 0.01 ^b^	0.5 ± 0.05 ^a^	0.4 ± 0.03 ^a^	0.4 ± 0.01 ^a^	1.1 ± 0.02 ^c^	1.1 ± 0.01 ^e^	1.5 ± 0.08 ^d^
B+EA-InA	0.7 ± 0.01 ^a^	1.2 ± 0.03 ^a^	0.4 ± 0.02 ^a^	0.4 ± 0.03 ^bc^	0.2 ± 0.01 ^b^	0.2 ± 0.00 ^bc^	1.5 ± 0.07 ^b^	2.7 ± 0.04 ^a^	5.8 ± 0.18 ^a^

EA: ellagic acid; EA-InSc: semicrystalline inulin–EA microparticles; EA-InA: amorphous inulin–EA microparticles; C: carbohydrate matrix (sucrose + pectin); P: protein matrix (whey protein concentrate); L: lipid matrix (sunflower oil); B: blend matrix. Results are expressed as mean ± standard deviation of three experiments. Lowercase letters indicate statistical differences (*p* ≤ 0.05) among systems in each digestion phase within each physical state of the microparticles (semicrystalline and amorphous) for each antioxidant activity assay. Interestingly, EA-InSc and EA-InA incorporated into carbohydrate- and blend-based matrices achieved the highest AA values across the three assays, suggesting interactions between EA and pectin via weak hydrogen bonds. Polyphenol–carbohydrate interactions have been widely reported, involving mainly hydrogen bonding and hydrophobic interactions [51]. In this case, EA appears to be partially entrapped within the pectin network, leading to the formation of a pectin–EA complex. The dissociation of this complex during the intestinal phase would depend on the equilibrium established in the intestinal medium, thereby modulating EA release.

## 4. Conclusions

This study shows that the performance of encapsulated phenolic compounds depends on the combined influence of the microparticle physical state, the structural features of the phenolic compounds, and the composition of the food matrix. GA, due to its high-water solubility, required mainly protection from degradation, whereas EA, with poor solubility, benefited from the semicrystalline state of inulin to enhance intestinal release. These differences indicate that encapsulation strategies must be specifically adapted to each phenolic compound rather than generalized. Antioxidant activity results further revealed that the digestive phase strongly conditions the efficacy of phenolic compounds: GA exhibited maximum activity in the gastric stage, while EA showed its highest potential during intestinal digestion, particularly in semicrystalline formulations. The incorporation of microparticles into carbohydrate- and blend-based matrices demonstrated that food components can significantly modulate release and antioxidant responses, highlighting the importance of considering the real food context in which functional ingredients are consumed. From a methodological perspective, experimental design for encapsulation should not focus solely on encapsulation efficiency or crystallinity index but also incorporate release and bioactivity during digestion as response variables, since these determine the actual functional performance of the system. Beyond these findings, the results provide guidance for developing functional foods enriched with phenolic compounds. Spray-dried inulin microparticles constitute a versatile platform, but their successful application requires aligning the chemical nature of the bioactive compound with the expected digestive and matrix interactions.

## Figures and Tables

**Table 1:** Second-order central composite design to study the effect of ethanol percentage (EtOH) and inlet air temperature (T_e_) on the encapsulation of ellagic acid with inulin by spray drying, and analysis of variance (ANOVA) for crystallinity index (CI) and encapsulation efficiency (EE).

EtOH (%)	Te (°C)	CI (%)	EE (%)
10	120	5.8 ± 0.1	86.0 ± 0.2
40	120	24.6 ± 1.8	81.4 ± 0.5
10	180	1.0 ± 0.1	77.6 ± 1.2
40	180	11.3 ± 0.2	57.6 ± 1.9
7	150	1.8 ± 0.3	87.5 ± 1.0
43	150	19.7 ± 0.5	63.4 ± 2.3
25	114	15.3 ± 0.8	82.1 ± 0.7
25	186	1.4 ± 0.1	73.2 ± 0.5
25	150	4.8 ± 0.1	77.8 ± 0.0
25	150	4.4 ± 0.4	83.0 ± 2.3
25	150	4.7 ± 0.6	80.9 ± 0.4
25	150	5.4 ± 0.3	76.7 ± 1.3
ANOVA crystallinity index			
Effect		Estimate	*p*-value
Intercept		4.9	
EtOH		14.65	0.0000 *
Te		−10.08	0.0001 *
EtOH^2^		7.62	0.0003 *
EtOH × Te		−4.25	0.002 *
Te^2^		4.34	0.0017 *
Lack-of-fit		0.0927	
R^2^ (%)		99.5	
R^2^ adjusted for degrees of freedom (%)		99	
ANOVA encapsulation efficiency			
Effect		Estimate	*p*-value
Intercept		78.85	
EtOH		−15.52	0.0058 *
Te		−12.4	0.0109 *
EtOH^2^			0.1426
EtOH x Te			0.0755
Te^2^			0.4363
Lack-of-fit		0.1894	
R^2^ (%)		79.1	
R^2^ adjusted for degrees of freedom (%)		74.5	

EtOH: Ethanol; T_e_: inlet air temperature to the dryer; EE: encapsulation efficiency; CI: Crystallinity index. The results are expressed as average ± standard deviation. *: significant variable (*p* ≤ 0.05).

**Table 2:** Characterization of semicrystalline and amorphous EA- and GA-inulin microparticles obtained by spray drying.

	EA-InSc	EA-InA	GA-InSc	GA-InA
Drying conditions				
T_a_ (°C)	20	20	20	20
T_e_ (°C)	114	148	114	148
Ethanol (%)	36.5	6.8	36.5	6.8
Microparticle characterization				
Total GA (mg/g)	-	-	9.6 ± 0.0 ^a^	9.5 ± 0.1 ^a^
Total EA (mg/g)	9.7 ± 0.1 ^a^	9.6 ± 0.1 ^a^	-	-
Yield	94.5 ± 0.5 ^aA^	91.7 ± 0.9 ^bA^	93.5 ± 0.5 ^aA^	91.6 ± 0.6 ^bA^
CI (%)	23.5 ± 0.5 ^aA^	2.1 ± 0.1 ^bA^	20.0 ± 0.02 ^aB^	1.7 ± 0.01 ^bB^
EE (%)	82.6 ± 0.7 ^aB^	83.4 ± 0.1 ^aB^	98.9 ± 0.02 ^bA^	99.2 ± 0.04 ^bA^
D_4,3_ (µm)	3.8 ± 0.4 ^aA^	3.3 ± 0.3 ^aA^	3.6 ± 0.2 ^aA^	3.2 ± 0.4 ^aA^

EA: Ellagic acid; GA: Gallic acid; InSc semicrystalline inulin; InA: amorphous inulin; T_a_: Infeed temperature; T_e_: inlet air temperature; IC: crystallinity index; EE: encapsulation efficiency. The results are expressed as average ± standard deviation. Different lowercase letters indicate significant differences (*p* ≤ 0.05) between semicrystalline and amorphous microparticle systems with the same polyphenol. Different capital letters indicate significant differences (*p* ≤ 0.05) between polyphenols for the same physical state (semicrystalline or amorphous).

## Data Availability

Data will be available on request.

## Abbreviations

The following abbreviations are used in this manuscript:

EA	Ellagic acid
GA	Gallic acid
In	Inulin
EtOH	Ethanol
Te	Inlet air temperature to the dryer
EE	Encapsulation efficiency
CI	Crystallinity index
SEM	Scanning electron microscopy
D4,3	Volume-weighted mean diameter
XRD	X-ray diffraction
InSc	Semicrystalline inulin
InA	Amorphous inulin
EA-InSc	Ellagic acid–inulin semicrystalline microparticles
EA-InA	Ellagic acid–inulin amorphous microparticles
GA-InSc	Gallic acid–inulin semicrystalline microparticles
GA-InA	Gallic acid–inulin amorphous microparticles
C	Carbohydrate matrix
P	Protein matrix
L	Lipid matrix
B	Blend matrix
AA	Antioxidant activity
DPPH	2,2-diphenyl-1-picrylhydrazyl
ABTS	2,2-azino-bis-3-ethylbenzothiazoline-6-sulphonic acid
